# The joint impact of elevated homocysteine and type 2 diabetes on non-healing wounds and all-cause mortality: integrated clinical and multi-omics analyses

**DOI:** 10.1097/JS9.0000000000003715

**Published:** 2025-11-04

**Authors:** Yichuan Li, Ying Wang, Heao Zhang, Xiaohang Xie, Lei Qin, Yuyang Zeng, Qi Zhang, Yong Zhang

**Affiliations:** a Department of Dermatology, Tongji Hospital, Tongji Medical College, Huazhong University of Science and Technology, Wuhan, China; b Department of Plastic and Cosmetic Surgery, Tongji Hospital, Tongji Medical College, Huazhong University of Science and Technology, Wuhan, China

**Keywords:** diabetes, homocysteine, mortality, NHANES, non-healing wounds, single-cell sequencing

## Abstract

**Background:**

Hyperhomocysteinemia (HHcy) and type 2 diabetes (T2D) are recognized risk factors for non-healing wounds, yet their combined effects and underlying mechanisms remain unclear.

**Methods:**

In this study of 8406 National Health and Nutrition Examination Survey participants (1999–2004), weighted logistic regression was used to estimate odds ratios for non-healing wounds and Cox proportional hazards models were employed to assess hazard ratios for all-cause mortality. Additive and multiplicative interactions were evaluated. Nonlinear relationships used restricted cubic splines. Mediation analyses explored inflammatory marker contributions. Bulk transcriptomic and single-cell sequencing data were integrated to identify homocysteine (Hcy)-diabetic foot ulcers (DFU) hub genes, construct an Hcy risk gene scoring system, and elucidate key mechanisms by which Hcy remodels the DFU microenvironment.

**Results:**

Both T2D [odds ratio (OR): 2.42, 95% confidence interval (CI): 1.69–3.47] and HHcy (OR: 1.67, 95% CI: 1.10–2.54) independently elevated non-healing wound risk. Participants with both conditions exhibited higher odds (OR: 5.28, 95% CI: 3.20–8.70), with additive interaction (relative excess risk due to interaction: 2.83, AP: 0.54, SI: 2.95). In diabetic men, Hcy displayed a J-shaped relationship with non-healing wounds (*P* for nonlinearity = 0.027), with the lowest risk around 8.9 µmol/L. In diabetic patients, inflammatory markers mediated the link, with proportions of 11.1% for red cell distribution width, 9.16% for monocyte/lymphocyte ratio, 5.28% for systemic inflammation response index, and 4.03% for neutrophil/lymphocyte ratio. Hcy drove immune-metabolic reprogramming in the DFU microenvironment by regulating key gene networks such as IL1B and MMP9, activating the MIF-CD74/CXCR4 axis to form a B cell-centric inflammatory cascade network. Both T2D and HHcy were associated with higher all-cause mortality, without joint interaction observed. Participants with a history of non-healing wounds had a higher all-cause mortality risk.

**Conclusions:**

T2D and HHcy synergistically worsen non-healing wounds, but not all-cause mortality. In diabetic males, Hcy levels displayed a J-shaped relationship with non-healing wound risk. Mechanistically, Hcy amplified systemic inflammation and drove stromal-immune crosstalk via the MIF-CD74/CXCR4 axis, reshaping the diabetic wound microenvironment.

## Introduction

Wound healing is a highly complex and tightly coordinated biological process involving multiple tissues and cells working together to repair injured structures and restore skin barrier function. Typically, wound healing progresses through four overlapping phases: hemostasis, inflammation, proliferation, and remodeling. However, non-healing wounds remain a persistent clinical challenge, affecting approximately 2.5% of the global population annually and incurring substantial economic losses on healthcare systems worldwide^[1]^. Non-healing wounds, including diabetic foot ulcers (DFUs), leg venous ulcers, arterial ulcers, pressure ulcers, and non-healing surgical wounds, fail to heal through normal physiological processes^[2]^. Non-healing wounds are often driven by complex pathophysiological comorbidities such as diabetes mellitus, obesity, metabolic disorders, vascular insufficiencies, and autoimmune diseases^[3]^.

Among individuals with diabetes, 19%–34% will develop DFUs during their lifetime, with 20% progressing to amputation and 10% experiencing mortality within the first year of DFU diagnosis^[4,5]^. Diabetic skin is often characterized by dryness, fragility, inflammatory cell infiltration, edema, and reduced granulation tissue formation, making it especially susceptible to infection and chronic non-healing wounds^[6]^. In a hyperglycemic (HG) environment, excessive formation of advanced glycation end products (AGEs) and reactive oxygen species (ROS) drives persistent inflammation and oxidative stress, impairing endothelial cells, fibroblasts, and keratinocytes, and disrupting the extracellular matrix. Concomitant alterations in the microvasculature further hinder oxygen and nutrient delivery, angiogenesis, and immune cell migration, collectively perpetuating a chronic wound microenvironment^[7]^. Chronic non-healing wounds drive the progressive accumulation of genetic and cellular damage through persistent injury, hyperactive repair mechanisms, or dysregulation of the wound healing pathway, ultimately leading to the development of inflammatory diseases, fibrosis, and even cancer^[8]^.

Homocysteine (Hcy) is a sulfur-containing, nonessential amino acid synthesized exclusively through the methionine (Met) cycle, with normal plasma levels ranging between 5 and 15 µmol/L^[9]^. Plasma levels exceeding 15 µmol/L define hyperhomocysteinemia (HHcy), which is recognized as an independent risk factor for deep vein thrombosis and peripheral arterial disease (PAD), possibly serving as a biomarker for lower-limb complications^[10]^. Mechanistically, elevated Hcy levels induce excessive ROS production, reduce nitric oxide (NO) bioavailability, and activate proinflammatory pathways, such as NF-κB and the inflammasome, thereby exacerbating oxidative stress and endothelial dysfunction. In diabetic patients, Hcy often remains chronically elevated due to impaired renal clearance, vitamin B12 and folate deficiencies, and disturbances in one-carbon metabolism^[11]^. When combined with hyperglycemia, HHcy further compromises endothelium-derived hyperpolarization factor (EDHF)-mediated vasodilation, heightens oxidative stress, and diminishes hydrogen sulfide (H_2_S) bioavailability, collectively exacerbating vascular and endothelial injury in T2D^[12]^.

This study aimed to investigate the synergistic and independent effects of elevated Hcy levels and T2D on the risk of non-healing wounds and all-cause mortality, using data from 8406 adults enrolled in the 1999–2004 National Health and Nutrition Examination Survey (NHANES) and linked to mortality data through 2019. We employed a comprehensive analytical approach that included weighted logistic regression, Cox proportional hazards models, and interaction analysis to assess the associations with non-healing wounds and mortality. This study also explored nonlinear relationships using restricted cubic splines and evaluated the mediating role of systemic inflammation by mediation analyses. Finally, transcriptomic and single-cell sequencing data were integrated to identify Hcy-DFU hub genes, construct an Hcy risk gene scoring system, and elucidate key mechanisms by which Hcy remodels the DFU microenvironment through metabolic pathway and cellular interaction analyses.

**HIGHLIGHTS**

- Type 2 diabetes and hyperhomocysteinemia synergistically elevate non-healing wound risk.
- Diabetic males show a J-shaped association between homocysteine (Hcy) and non-healing wound risk.
- Inflammation mediates the association between Hcy and diabetic non-healing wounds.
- Multi-omics analyses uncover that Hcy drives immune-metabolic reprogramming in diabetic foot ulcers.
- A history of non-healing wounds contributes to a higher mortality risk.

## Materials and methods

### Study design and participants

This cross-sectional study utilized data from the NHANES conducted by the Centers for Disease Control and Prevention (CDC). All participants provided written informed consent at enrollment, and the NHANES study was approved by the Institutional Review Board of the National Center of Health Statistics. The work has been reported in line with the STROCSS criteria^[13]^.

**Figure 1.**
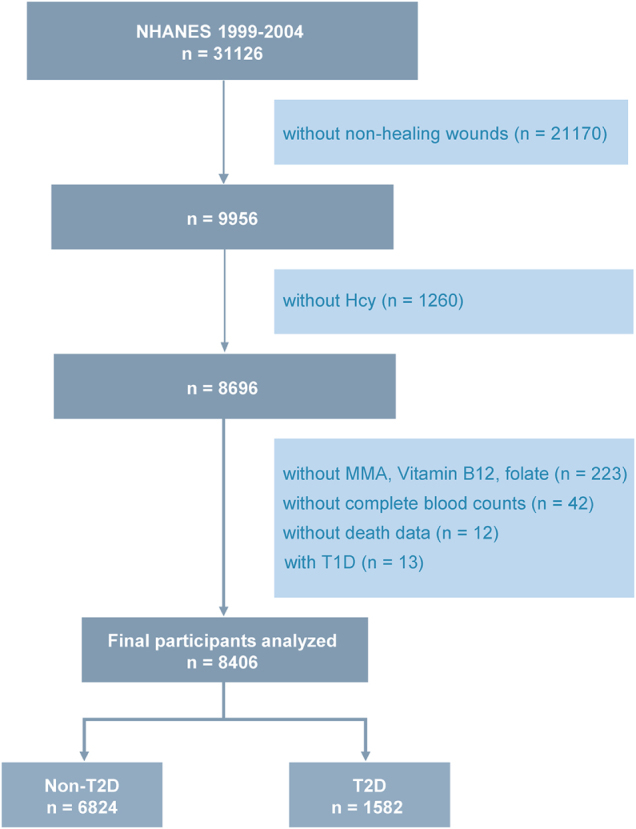
Flowchart of the sample selection from NHANES 1999–2004. NHANES, National Health and Nutrition Examination Survey.

In this analysis, we reviewed data from 31 126 participants collected during the NHANES cycles between 1999 and 2004. Participants with missing data on non-healing wounds (*n* = 21 170), plasma total Hcy (*n* = 1260), cobalamin-related biomarkers [methylmalonic acid (MMA), vitamin B12, and folate] (*n* = 223), complete blood counts (*n* = 42), or mortality information (*n* = 12) were excluded from the analysis. Additionally, individuals potentially with type 1 diabetes (T1D, defined as those aged <20 years who used only insulin) were also excluded (*n* = 13). T2D was defined as meeting any of the following criteria: self-reported doctor diagnosis of diabetes, use of insulin or oral hypoglycemic medication, fasting plasma glucose (FPG) ≥ 7.0 mmol/L, or glycated hemoglobin A1c (HbA1c) ≥ 6.5%. The final analytical sample included 8406 participants (1582 with T2D) (Fig. 1).

### Exposure and outcome variables

The main exposure variable was Hcy levels. Hcy levels were measured using the Abbott Homocysteine assay, a fully automated fluorescence polarization immunoassay performed on the Abbott IMx analyzer (1999–2001) and the Abbott Axsym analyzer (2002–2004), with a comparative study demonstrating no significant differences between these two methods^[14]^. HHcy was defined as Hcy levels >15 μmol/L.

The primary outcomes were the occurrence of non-healing wounds and all-cause mortality. Non-healing wounds were defined based on self-reported health interviews, whether ever had an ulcer or sore on the leg or foot that took more than 4 weeks to heal. Mortality data were obtained by linking the cohort database to the National Death Index (NDI) through 31 December 2019, and all-cause mortality was defined as death from any cause.

### Covariates

Sociodemographic data were collected via questionnaires, including age, sex (male or female), race (Mexican American, non-Hispanic White, non-Hispanic Black, or Other), marital status (married or living alone), and the family poverty-to-income ratio (PIR). Smoking status was categorized into three groups: never smokers (fewer than 100 cigarettes in their lifetime), former smokers (more than 100 cigarettes in their lifetime but not currently smoking), and current smokers (actively smoking). Alcohol status was classified as drinker (more than 12 alcohol drinks per year) or non-drinker (fewer than 12 alcohol drinks per year). Body mass index (BMI) was calculated as weight (kg) divided by height (m^2^). In addition, levels of HbA1c, total cholesterol (TC), high-density lipoprotein cholesterol (HDL), red cell distribution width (RDW), lactate dehydrogenase (LDH), C-reactive protein (CRP), vitamin B12, folate, MMA, and complete blood counts were measured at recruitment. Selected markers for mediation analysis include MLR (monocyte/lymphocyte ratio), NLR (neutrophil/lymphocyte ratio), SIRI (systemic inflammation response index: (neutrophils × monocytes)/lymphocytes), and RDW^[15]^.

### Ascertainment of mortality

Follow-up and mortality data were obtained by linking NHANES records to the NDI through 31 December 2019, with all-cause mortality defined as death from any cause.

### Transcriptomic analysis of Hcy and DFU-related genes

#### Identification of Hcy-related genes

We employed a multi-source database integration strategy to identify potential targets of Hcy. First, we utilized the GSE175735 dataset from the Gene Expression Omnibus (GEO) database, which includes transcriptome profiling data from three Hcy-treated aortic endothelial cell samples and three control samples^[16]^. Differential expression analysis identified 29 genes induced by Hcy treatment. Additionally, using “Homocysteine” as a keyword, we retrieved 249 predicted Hcy target genes from the Comparative Toxicogenomics Database (CTD) and 571 Hcy-related genes from the GeneCards database. By integrating these data sources, we ultimately identified 793 Hcy-related genes^[17,18]^.

#### Bulk transcriptomic data for DFU

We obtained three DFU-related gene expression matrices from the GEO database for transcriptomic analysis. Bulk RNA sequencing (RNA-seq) analysis was conducted using the GSE199939 dataset, which includes foot skin transcriptomic data from 10 DFU patients and 11 non-diabetic healthy controls^[19]^. Differential gene expression analysis was carried out using the “limma” package in R, with statistical significance thresholds set at *P* < 0.05 and |log2FC| > 1^[20]^. Data visualization was performed using the “ggplot2” and “pheatmap” packages to generate volcano plots and heatmaps, illustrating the overall expression differences between the DFU and control groups. Gene Set Enrichment Analysis (GSEA) was conducted using the “GseaVis” package in R^[21,22]^.

#### Integrative analysis and network construction

Venn diagrams were generated using the R package “ggvenn.” Gene Ontology (GO) and Kyoto Encyclopedia of Genes and Genomes (KEGG) pathway analyses were performed using the R package “clusterProfiler”^[23]^. The intersection between Hcy-related and DFU-related genes was imported into the STRING database to construct a protein–protein interaction (PPI) network model. The resulting PPI network was subsequently analyzed in Cytoscape 3.10.1 for topological characteristics. The “CytoNCA” plugin was used to calculate centrality metrics, including Betweenness, Closeness, Degree, Eigenvector, LAC, and Network. Hub genes were further prioritized using additional analysis plugins, such as BottleNeck, Closeness, Degree, EPC, MCC, MNC, Radiality, and Stress^[24]^. Transcription factor predictions were conducted using the “NetworkAnalyst” platform for analysis and visualization. The area under the receiver operating characteristic curve (AUC) was calculated using the R package “pROC” to evaluate the diagnostic value of the hub genes in DFU^[25]^.

### Single-cell RNA-seq analysis

To investigate cell-type-specific mechanisms in DFUs, we performed single-cell transcriptomic analysis using the publicly available GEO dataset GSE165816, which includes single-cell RNA-seq profiles from five DFU tissue samples and eight non-ulcerated diabetic foot skin samples^[26]^.

#### Data processing and quality control

Data were processed using the “Seurat” R package^[26]^. Initial quality control involved filtering out genes detected in fewer than five cells and cells expressing fewer than 300 genes. Cells were further retained based on thresholds of 500–5000 detected genes, total UMI counts <10 000, mitochondrial gene content <20%, ribosomal gene content <3%, and hemoglobin gene content >1%. Ambient RNA contamination was computationally removed using “decontX,” and doublets were identified and excluded via “DoubletFinder”^[27,28]^.

#### Integration and clustering

Normalization was performed using log normalization, followed by the selection of the top 2000 highly variable genes. Cell cycle scores were calculated, and data scaling was applied to regress out technical variations. Dimensionality reduction was performed using principal component analysis (PCA), with batch effect correction via the “Harmony” algorithm. UMAP and Louvain clustering were applied using the first 20 principal components at a resolution of 0.2^[29]^.

#### Definition of STARTRAC indices for tissue distribution

To evaluate the tissue distribution preferences of cell clusters, we computed the STARTRAC-dist index, which quantifies enrichment using the ratio of observed to expected cell frequencies (*R*_o/e_).

$$I_{dist}^{STARTRAC}=R_{o/e}=\frac{observed}{expected}$$

For each combination of cell cluster and tissue, observed cell counts were compared to expected counts under the assumption of random distribution, based on the chi-squared test. An *R*_o/e_ > 1 indicates enrichment, while *R*_o/e_ < 1 indicates depletion of the given cluster in the tissue^[30]^.

Additionally, odds ratios (OR) were calculated to further characterize tissue distribution preferences. Clusters with OR > 1.5 were considered enriched in a tissue, whereas those with OR < 0.5 were considered depleted^[31]^.

#### Single-cell Hcy risk scoring

To quantify the regulatory activity of Hcy-related genes at the single-cell level, we applied the R package “CRDscore”^[32–34]^. Briefly, we first extracted the raw count expression matrix *M* from the scRNA-seq data and computed the mean expression level *E_i_* for each gene *i*. Then, using a random sampling approach, all genes were sorted into 50 expression bins based on their *E_i_*, and the frequency of occurrence of Hcy-related genes, designated as *B_f_*, was calculated. Random feature genes were selected from each bin based on the *B_f_* count from random sampling, with the overall quantity of random feature genes being equal to the number of Hcy-related genes. This random process was iterated 50 times. Lastly, a gene-centered expressed matrix was constructed, which can be read as data devoid of excessive migratory signals:

$$Z_{i,j}=M_{i,j}-\frac{\left(\sum_{j}M_{i,j}\right)}{N}$$

Next, for each cell, a centered expression score was calculated using a centralized expression matrix, where each gene expression was adjusted by subtracting its mean expression across all cells. The final “CRDscore” was defined as the difference between the centered expression of the input genes and the average of background scores. Cells were then classified into Hcy-High and Hcy-Low groups according to the median risk score.

#### Gene set enrichment analysis

Single-cell gene set enrichment analysis was performed using the “irGSEA” R package on the RNA assay, applying both “AUCell” and “UCell” methods to quantify pathway activity at the single-cell level^[35]^. Gene sets were derived from the Hallmark collection of MSigDB. To identify pathways consistently enriched across methods, Robust Rank Aggregation was used to integrate results. Gene sets with an adjusted *P*-value ≤ 0.05 were considered significant. A total of seven significantly enriched pathways were identified and visualized.

Cellular metabolic activity was assessed using a gene set-based weighted scoring strategy. Eighty-five metabolic pathways from the KEGG database were filtered (retaining pathways with ≥ 5 overlapping genes), and pathway-specific activity scores were calculated for each cell^[36]^.

#### Cell–cell communication

Cell–cell communication was analyzed with “CellChat,” which integrates single-cell transcriptomic data to quantify ligand–receptor pairs and identify key regulatory units in signaling networks^[37]^.

### Statistical analysis

#### Complex survey design and descriptive statistics

NHANES data use a complex, multi-stage sampling design with primary sampling units, strata, and weights to ensure nationally representative estimates. Continuous variables are presented as mean [standard deviation (SD)] and were compared using the design-based Kruskal–Wallis test. Categorical variables were expressed as unweighted counts (weighted proportions) and were compared using the Rao & Scott adjusted Pearson’s chi-square test.

#### Regression and survival analyses

The association between elevated Hcy and T2D with non-healing wounds was analyzed using weighted logistic regression models. Similarly, the relationship between elevated Hcy and T2D with all-cause mortality, both in the overall population and among participants with non-healing wounds, was evaluated using weighted Cox proportional hazards models. Both models were adjusted for key covariates, including age, sex, race, marital status, smoking status, alcohol consumption, family PIR, BMI, vitamin B12, and MMA.

#### Interaction analysis

The interaction terms were assessed using the “interaction” package. For additive interaction, three indices were computed: the relative excess risk due to interaction (RERI), the attributable proportion due to interaction (AP), and the synergy index (SI), with 95% confidence intervals estimated via the delta method^[38]^. Multiplicative interaction was assessed using likelihood ratio tests^[39]^.

#### Nonlinear and survival curve analysis

Restricted cubic spline analysis, with four knots at the 5th, 35th, 65th, and 95th percentiles of Hcy distribution, was applied to explore the nonlinear relationship between Hcy levels and non-healing wounds. Kaplan–Meier curves visualized mortality rates across groups, with comparisons conducted via log-rank tests.

#### Mediation analysis

A three-variable mediation model was constructed using the “mediation” package in R to quantify the extent to which inflammatory markers mediate the association between Hcy and non-healing wounds in diabetic individuals^[40]^. The significance of the mediation effects was assessed using 5000 bootstrap iterations. This analysis yielded estimates of the total effect, direct effect, and mediation effect, with the total effect defined as the sum of the direct and indirect (mediation) effects^[41–43]^. The mediation proportion was calculated as the percentage of the total effect attributable to the mediator, showing how much the inflammatory marker explained the observed relationship.

Survey weights were not applied in the Kaplan–Meier, restricted cubic spline, and mediation analyses due to the limited number of non-healing wound cases, which could have led to instability in the estimates and compromised the reliability of the results.

#### Sensitivity analyses

Sensitivity analyses were performed to assess the robustness of the results. First, synergistic effects of T2D and HHcy on the risk of non-healing wounds were explored using unweighted HHcy thresholds (Hcy >15 µmol/L) and weighted analyses using tighter thresholds (Hcy >10 µmol/L). Second, quintile and stratified analyses confirmed the association between Hcy and non-healing wounds in diabetic patients. Finally, to further validate the generalizability of the J-shaped relationship, an RCS analysis was performed in the general male population.

#### Handling of missing data and statistical significance

Variables with missing values exceeding 10% were excluded, and the remaining missing data were imputed using the random forest algorithm via the “missForest” package. A two-sided *P* < 0.05 was considered significant. All analyses were performed with R version 4.3.2.

## Results

### Baseline characteristics of all participants

A total of 8406 participants were enrolled in the study, including 1582 individuals with T2D (weighted, 13.62%). Among the participants, the mean age was 56.84 years, 46.96% being male; 1795 (4.71%) were Mexican American, 4529 (77.17%) were non-Hispanic White, 1499 (9.28%) were non-Hispanic Black, and 583 (8.83%) were categorized as Other. The baseline characteristics, grouped by the presence of non-healing wounds (319 with non-healing wounds; 8087 without non-healing wounds), are summarized in Table 1.

**Table 1:** Baseline characteristics of all participants with or without non-healing wounds

Characteristic	Overall (*n* = 8406)	Without non-healing wounds (*n* = 8087)	With non-healing wounds (*n* = 319)	*P* value
Age	56.84 (12.61)	56.74 (12.59)	60.02 (12.77)	<0.001
Sex				0.072
Male	4155 (46.96%)	3983 (46.77%)	172 (52.77%)	
Female	4251 (53.04%)	4104 (53.23%)	147 (47.23%)	
Race				0.335
Mexican American	1795 (4.71%)	1722 (4.71%)	73 (4.69%)	
Non-Hispanic White	4529 (77.17%)	4361 (77.19%)	168 (76.68%)	
Non-Hispanic Black	1499 (9.28%)	1440 (9.22%)	59 (11.35%)	
Other	583 (8.83%)	564 (8.88%)	19 (7.27%)	
Marital status				0.011
Married	5222 (66.51%)	5055 (66.83%)	167 (56.36%)	
Living alone	3184 (33.49%)	3032 (33.17%)	152 (43.64%)	
Alcohol status				0.656
Drinker	2882 (31.12%)	2766 (31.08%)	116 (32.38%)	
Non-drinker	5524 (68.88%)	5321 (68.92%)	203 (67.62%)	
Smoking status				<0.001
Never smoker	3962 (47.05%)	3,842 (47.42%)	120 (35.80%)	
Former smoker	2862 (32.78%)	2749 (32.78%)	113 (32.73%)	
Current smoker	1582 (20.17%)	1496 (19.80%)	86 (31.47%)	
Family PIR	3.15 (1.54)	3.17 (1.54)	2.45 (1.47)	<0.001
BMI	28.59 (6.08)	28.52 (5.99)	30.77 (7.96)	<0.001
Waist	98.96 (14.77)	98.75 (14.60)	105.55 (17.95)	<0.001
HbA1C	5.62 (0.98)	5.61 (0.96)	6.08 (1.43)	<0.001
TC	5.43 (1.03)	5.44 (1.03)	5.26 (1.09)	0.012
HDL	1.37 (0.42)	1.37 (0.42)	1.35 (0.48)	0.067
RDW	12.75 (1.16)	12.74 (1.15)	13.12 (1.17)	<0.001	
CRP	0.47 (0.86)	0.46 (0.84)	0.72 (1.25)	<0.001	
LDH	139.40 (31.92)	139.20 (31.81)	145.47 (34.68)	0.018	
Folate	36.70 (32.77)	36.62 (32.13)	39.15 (48.65)	0.313	
Vitamin B12	404.33 (970.58)	402.04 (961.48)	474.90 (1218.18)	0.200	
MMA	171.96 (201.22)	171.20 (202.64)	195.28 (149.39)	0.003	
Diabetes status				<0.001	
Non-diabetes	6824 (86.38%)	6629 (87.02%)	195 (66.48%)		
Diabetes	1582 (13.62%)	1458 (12.98%)	124 (33.52%)		
Hcy	9.44 (4.49)	9.39 (4.38)	11.11 (6.94)	<0.001	
Hcy status				<0.001	
Non-HHcy	7737 (94.09%)	7463 (94.31%)	274 (87.27%)		
HHcy	669 (5.91%)	624 (5.69%)	45 (12.73%)		
Diabetes/HHcy Group				<0.001	
Non-diabetes/Non-HHcy	6339 (81.81%)	6161 (82.49%)	178 (60.84%)		
Non-diabetes/HHcy	485 (4.57%)	468 (4.53%)	17 (5.64%)		
Diabetes/Non-HHcy	1398 (12.29%)	1302 (11.83%)	96 (26.44%)		
Diabetes/HHcy	184 (1.34%)	156 (1.15%)	28 (7.08%)		

BMI, body mass index; CRP, C-reactive protein; HbA1C, hemoglobin type A1C; Hcy, homocysteine; HDL, high-density lipoprotein cholesterol; HHcy, hyperhomocysteinemia; LDH, lactate dehydrogenase; MMA, methylmalonic acid; PIR, poverty-to-income ratio; RDW, red cell distribution width; TC, total cholesterol; TC, total cholesterol.

Data were adjusted for complex survey designs, and the observed numbers for categorical variables were unweighted.

Participants with non-healing wounds were generally older, more likely to be male, current smokers, and living alone. They also had a lower PIR and TC, but higher BMI, waist circumference, HbA1c, RDW, CRP, LDH, and MMA, compared to those without wounds. Additionally, participants with non-healing wounds had significantly elevated Hcy levels, a greater prevalence of diabetes, a higher frequency of hyperhomocysteinemia (HHcy, Hcy > 15 µmol/L), and a larger proportion belonged to the combined diabetes/HHcy group. No significant differences were observed between the two groups in terms of race, alcohol consumption, HDL, folate, and vitamin B12. Further baseline characteristics of the total population, stratified by diabetes/HHcy groups and mortality status, are provided in Supplemental Digital Content Table S1 and S2, available at: http://links.lww.com/JS9/F425.

### Association and interaction between T2D and Hcy levels on non-healing wounds

The weighted multivariable logistic regression models were used to investigate the association between T2D and elevated Hcy levels with non-healing wounds (Table 2). After multivariable adjustment, each 1 µmol/L increase in Hcy was associated with a 4% greater risk of non-healing wounds (OR: 1.04, 95% CI: 1.01–1.06, *P* **=** 0.002). Additionally, both HHcy and T2D independently contributed to the elevated risk, with ORs of 1.67 (95% CI: 1.10–2.54, *P* = 0.017) and 2.42 (95% CI: 1.69–3.47, *P* < 0.001), respectively.

**Table 2:** Logistic regression analysis of the associations between T2D and elevated Hcy with the risk of non-healing wounds in all participants

Variable	Crude model		Model 1		Model 2	
	OR (95% CI)	*P* value	OR (95% CI)	*P* value	OR (95% CI)	*P* value
Continuous						
Hcy	1.04 (1.02–1.06)	**<0.001**	1.03 (1.02–1.05)	**<0.001**	1.04 (1.01–1.06)	**0.002**
Group						
Non-HHcy	Ref.		Ref.		Ref.	
HHcy	2.42 (1.65–3.54)	**<0.001**	1.87 (1.23–2.85)	**0.005**	1.67 (1.10–2.54)	**0.017**
Non-diabetes	Ref.		Ref.		Ref.	
Diabetes	3.38 (2.51–4.56)	**<0.001**	3.07 (2.25–4.20)	**<0.001**	2.42 (1.69–3.47)	**<0.001**
Diabetes/HHcy Group						
Non-diabetes/Non-HHcy	Ref.		Ref.		Ref.	
Non-diabetes/HHcy	1.69 (0.98–2.89)	0.057	1.39 (0.8–2.44)	0.2	1.24 (0.71–2.17)	0.4
Diabetes/Non-HHcy	3.03 (2.11–4.35)	**<0.001**	2.81 (1.94–4.07)	**<0.001**	2.21 (1.44–3.38)	**<0.001**
Diabetes/HHcy	8.35 (5.10–13.66)	**<0.001**	6.68 (3.87–11.53)	**<0.001**	5.28 (3.20–8.70)	**<0.001**

BMI, body mass index; CI, confidence interval; HHcy, hyperhomocysteinemia; MMA, methylmalonic acid; OR, odds ratio.

Model 1: Adjusted for age, sex, race, and marital status. Model 2: Adjusted for age, sex, race, marital status, smoking status, alcohol use, family poverty-to-income ratio, BMI, vitamin B12, and MMA. All estimates accounted for complex survey designs. Bold values indicate statistical significance at *P* < 0.05.

In joint analyses, compared with the non-diabetes/non-HHcy group, the ORs for non-healing wounds in the non-diabetes/HHcy, diabetes/non-HHcy, and diabetes/HHcy groups were 1.24 (95% CI: 0.71–2.17, *P* = 0.4), 2.21 (95% CI: 1.44–3.38, *P* < 0.001), and 5.28 (95% CI: 3.20–8.70, *P* < 0.001), respectively (Table 2).

Additionally, interaction analyses were conducted to evaluate both additive (RERI, AP, and SI) and multiplicative interactions between T2D and HHcy on the risk of non-healing wounds (Table 3). On the additive scale, a significant positive interaction was observed (RERI: 2.83, 95% CI: 0.22–5.44), indicating that beyond the sum of their individual effects, T2D and HHcy together contributed an excess risk of 2.83. Additionally, 54% of the excess risk among individuals jointly exposed to T2D and HHcy was attributable to their interaction (AP: 0.54, 95% CI: 0.25–0.82). Furthermore, the combined effect of T2D and HHcy was 2.95 times the sum of their individual effects (SI: 2.95, 95% CI: 1.17–7.43). However, no significant multiplicative interaction was detected (OR: 1.92, 95% CI: 0.89–4.14, *P* = 0.11).

**Table 3:** Additive and multiplicative interaction analysis of T2D and HHcy on the risk of non-healing wounds in all participants

Population	Additive interactive (95% CI)		Multiplicative interactive OR (95%CI)	*P* value
All participants	RERI	2.83 (0.22, 5.44)	1.92 (0.89, 4.14)	0.11
	AP	0.54 (0.25, 0.82)		
	SI	2.95 (1.17, 7.43)		

AP, attributable proportion; MMA, methylmalonic acid; RERI, relative excess risk due to interaction; SI, synergy index;

The multivariable-adjusted model additionally adjusted for age, sex, race, marital status, smoking status, alcohol use, family poverty-to-income ratio, BMI, Vitamin B12, and MMA. All estimates accounted for complex survey designs.

### Specific analysis of Hcy with non-healing wounds in diabetic patients

The baseline characteristics of the 1582 diabetic participants, categorized by the presence of non-healing wounds and Hcy levels, are presented in Supplemental Digital Content Table S3 and S4, available at: http://links.lww.com/JS9/F425. Logistic regression models were employed to investigate the association between Hcy and non-healing wounds in diabetic patients (Supplemental Digital Content Table S5, available at: http://links.lww.com/JS9/F425). In the fully adjusted Model 2, each 1 µmol/L increase in Hcy was linked to a 7% greater risk of non-healing wounds (OR: 1.07, 95% CI: 1.01–1.14, *P* = 0.017). Moreover, participants with HHcy also showed an increased risk after adjustments (OR: 2.65, 95% CI: 1.39–5.03, *P* = 0.004).

#### Subgroup analysis

**Figure 2.**
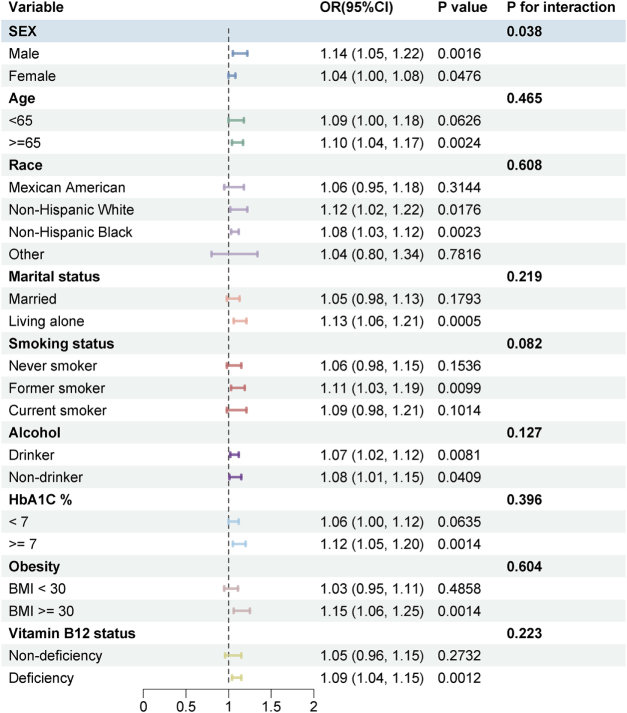
Subgroup analysis of the associations between Hcy and sex with the risk of non-healing wounds in diabetic patients. The model was adjusted for age, sex, race, marital status, smoking status, alcohol use, family PIR, BMI, vitamin B12, and methylmalonic acid. For subgroup analyses, the stratifying variable was not included as a covariate in the corresponding models. BMI, body mass index; CI, confidence interval; OR, odds ratio; PIR, poverty-to-income ratio.

Stratified analyses indicated a significant interaction between Hcy and sex with the risk of non-healing wounds in diabetic patients (*P* = 0.038 for interaction, Fig. 2).

No significant nonlinear association was observed for the restricted cubic spline (RCS) curve among all diabetic patients (*P* for nonlinearity = 0.231) and female diabetic patients (*P* for nonlinearity = 0.321) (Supplemental Digital Content Figure S1, available at: http://links.lww.com/JS9/F425.

**Figure 3.**
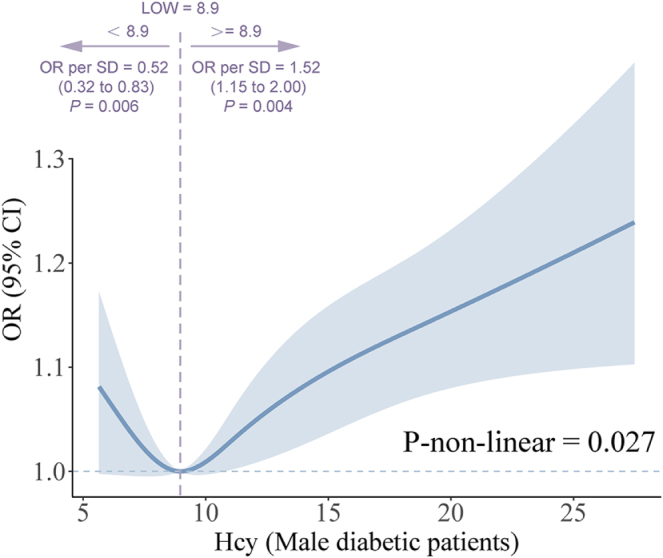
Association of Hcy levels with the risk of non-healing wounds in male diabetic patients by restricted cubic splines. The reference point was determined as the Hcy value associated with the lowest odds ratio (OR), with knots placed at the 5th, 35th, 65th, and 95th centiles of the Hcy distribution. ORs are indicated by solid lines and 95% CIs by shaded areas. The model was adjusted for age, race, marital status, smoking status, alcohol use, family poverty-to-income ratio, BMI, vitamin B12, and methylmalonic acid. CI, confidence interval; OR, odds ratio.

However, a J-shaped relationship between Hcy levels and non-healing wounds was identified in male diabetic patients (Fig. 3), with the lowest risk at approximately 8.9 µmol/L (*P* for nonlinearity = 0.027). Below 8.9 µmol/L, each standard deviation increase in Hcy was linked to a 48% reduction in the risk of non-healing wounds (OR: 0.52, 95% CI: 0.32–0.83, *P* = 0.006), whereas above it, each standard deviation increase was associated with a 52% increase in risk (OR: 1.52, 95% CI: 1.15–2.00, *P* = 0.004).

#### Mediation analysis

**Figure 4.**
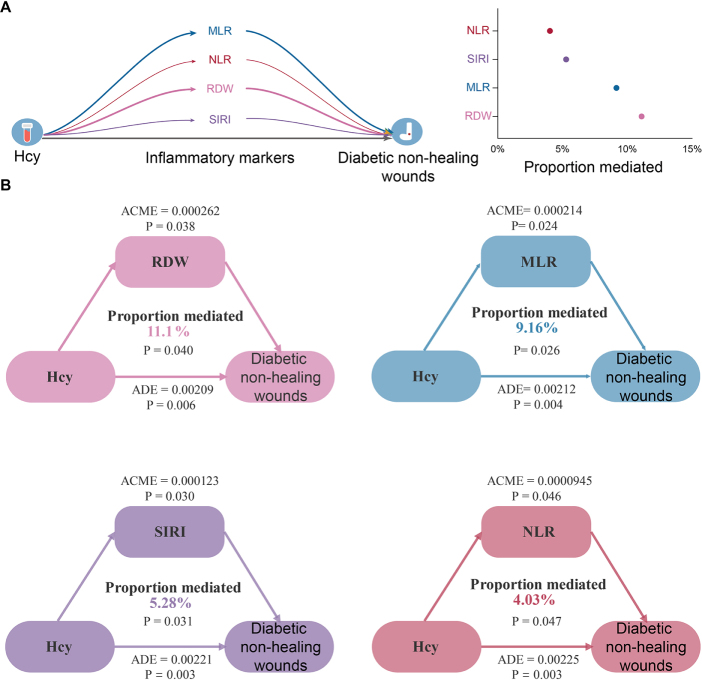
Mediation analysis of inflammatory markers in the association between Hcy and non-healing wounds in diabetic patients. (A) MLR, NLR, RDW, and SIRI significantly mediated the prospective association between Hcy and non-healing wounds in diabetic patients. The thickness of the path indicates the strength of the association, and numerical values for the mediating effect sizes are provided. Dots: mediation ratio. (B) Detailed mediation analysis of inflammatory markers in the association between Hcy and non-healing wounds in diabetic patients. Arrows indicate promotion. ACME, average causal mediation effects; ADE, average direct effects. All models were adjusted for age, sex, race, marital status, smoking status, alcohol use, family poverty-to-income ratio, BMI, vitamin B12, and methylmalonic acid. BMI, body mass index; MLR,monocyte/lymphocyte ratio; NLR, neutrophil/lymphocyte ratio; RDW, red cell distribution width; SIRI, systemic inflammation response index.

Mediation analyses demonstrated a partial but significant mediation effect of inflammatory markers on the association between Hcy and non-healing wound incidence in diabetic patients, independent of all covariates. Specifically, the proportions mediated were 11.1% for RDW, 9.16% for MLR, 5.28% for SIRI, and 4.03% for NLR (Fig. 4).

### Sensitivity analyses

To further assess the robustness of our results, we initially performed an unweighted interaction analysis using the HHcy threshold of >15 µmol/L. The findings confirmed that the synergistic effect between T2D and HHcy on the risk of non-healing wounds remained significant (Supplemental Digital Content Table S6, available at: http://links.lww.com/JS9/F425). Given that studies have shown even mildly elevated Hcy levels (>10 µmol/L) are associated with increased risks of cardiovascular and neurodegenerative diseases, we subsequently adjusted the HHcy threshold to >10 µmol/L in the weighted interaction analysis, which further reinforced the synergistic effect between T2D and HHcy on non-healing wounds (Supplemental Digital Content Table S7, available at: http://links.lww.com/JS9/F425). Additionally, quintile analysis confirmed the association between Hcy and non-healing wounds in diabetic patients. Participants in the fifth quintile (Q5) exhibited a significantly higher risk compared to those in the first quintile (Q1), with a notable trend across all quintiles (Supplemental Digital Content Table S8, available at: http://links.lww.com/JS9/F425). To further verify the generalizability of the J-shaped Hcy-risk relationship, RCS analysis was performed on the general male population. The risk of non-healing wounds was lowest at approximately 8.8 µmol/L (Supplemental Digital Content Figure S2, available at: http://links.lww.com/JS9/F425,

### Molecular mechanisms underlying Hcy in diabetic non-healing wounds

#### The overall expression profile in DFU

**Figure 5.**
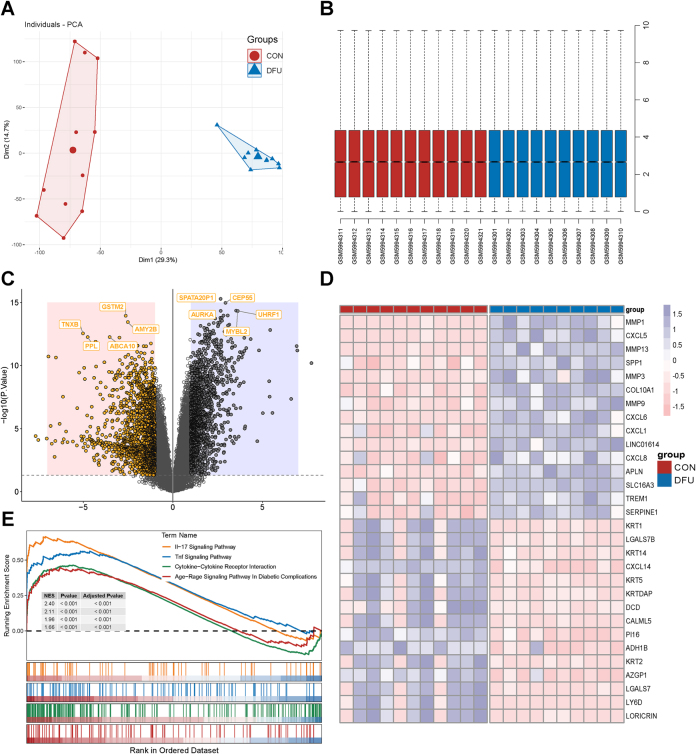
Analysis of the DFU dataset GSE199939. (A) PCA plots showing clustering of CON and DFU groups based on gene expression profiles. (B) Boxplots of normalized expression. (C) Volcano plot of DEGs. (D) Heatmap of the top 15 upregulated and top 15 downregulated DEGs. (E) GSEA Pathway Enrichment. CON, control; DEGs, differentially expressed genes; DFU, diabetic foot ulcer; GSEA, Gene Set Enrichment Analysis; PCA, principal component analysis.

We collected transcriptome data for 10 DFU patients and 11 healthy controls from the dataset GSE199939. Following gene expression normalization and batch effect correction, we conducted a PCA and identified two distinct clustering patterns, indicating overall gene expression differences between the DFU and control (CON) groups (Fig. 5A and B). Analysis of differentially expressed genes (DEGs) revealed 1541 upregulated and 1632 downregulated genes (Fig. 5C and D). These DEGs were further subjected to GSEA, which revealed that inflammatory pathways, including “IL-17 Signaling Pathway,” “TNF Signaling Pathway,” “Cytokine-Cytokine Receptor Interaction,” and “AGE-RAGE Signaling Pathway in Diabetic Complications,” were significantly enriched in DFU (Fig. 5E). The coordinated activation of these inflammation-associated pathways suggests a pivotal role of inflammatory dysregulation in DFU pathogenesis.

#### Hcy-associated gene signatures and key regulatory networks

**Figure 6.**
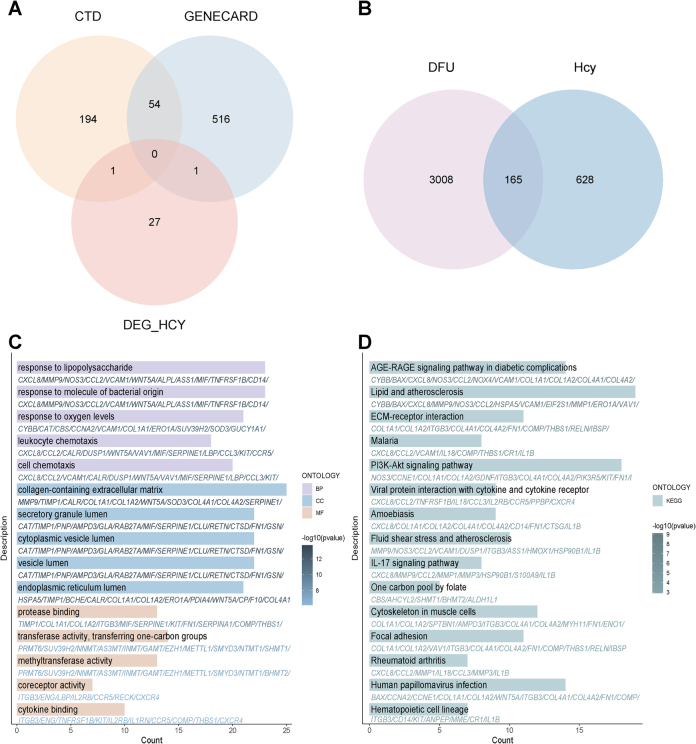
Identification of Hcy-related genes and biological pathway analysis. (A) The intersected potential targets from three databases used in the present study. (B) Intersection analysis between DFU-associated DEGs and Hcy target genes. (C) GO enrichment analysis. (D) KEGG enrichment analysis. DEGs, differentially expressed genes; DFU, diabetic foot ulcer; GO, Gene Ontology; KEGG, Kyoto Encyclopedia of Genes and Genomes.

To identify potential gene targets mediating the regulatory effects of Hcy on DFU pathogenesis, we constructed a comprehensive Hcy-DFU interaction network. About 249 Hcy target genes were obtained from the CTD database, 571 genes from the GeneCards database, and 29 DEGs from the GSE175735 dataset. Con-sequently, a total of 793 combined Hcy target genes from these databases were considered potential target genes of Hcy (Fig. 6A). In Fig. 6B, the Venn diagram highlights 165 overlapping genes implicated in Hcy-driven mechanisms underlying DFU progression. These overlapping genes were further subjected to GO analysis, which revealed significant alterations including “response to lipopolysaccharide,” “response to molecule of bacterial origin,” “response to oxygen levels,” and “leukocyte chemotaxis” (Fig. 6C). Additionally, our KEGG analysis demonstrated significant alterations in “AGE-RAGE signaling pathway in diabetic complications,” “Lipid and atherosclerosis,” “ECM-receptor interaction,” “IL-17 signaling pathway,” “One-carbon pool by folate,” and “Focal adhesion” (Fig. 6D).

**Figure 7.**
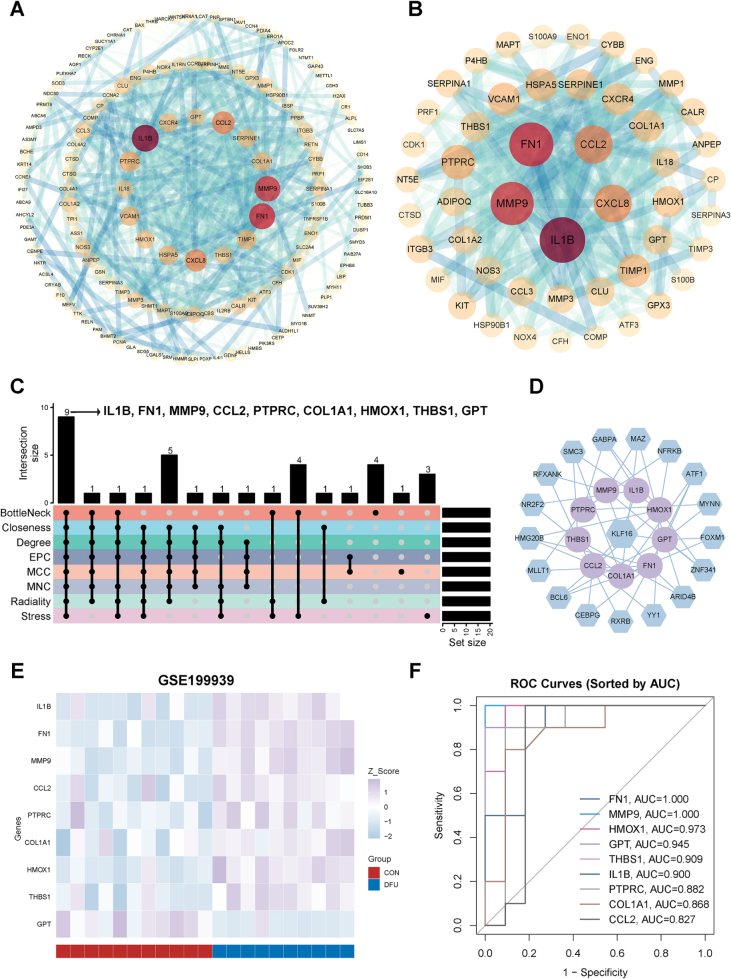
Integrated analysis of Hcy-related genes in DFU. (A) Integrated PPI network of overlapping 165 targets. (B) Top-ranked PPI subnetwork filtered by CytoNCA. (C) Core hub genes identified via 8 CytoHubba algorithms. (D) Transcriptional regulatory network of hub genes. (E) Heatmap of differential expression of hub genes in DFU and CON samples (GSE199939). (F)ROC curves of 9 genes for predicting the occurrence of the DFU. CON, control; DFU, diabetic foot ulcer; PPI, protein–protein interaction.

PPI and hub genes analyses of 165 overlapping genes emphasized the crucial roles of IL1B, FN1, and MMP9 as central hubs (Fig. 7A-B). The network essentiality of overlapping targets was assessed using eight algorithms (BottleNeck, Closeness, Degree, EPC, MCC, MNC, Radiality, and Stress). The top 20 proteins from each algorithm’s ranked list were selected as DFU candidate targets. Intersection analysis across all algorithms prioritized nine hub genes (IL1B, FN1, MMP9, CCL2, PTPRC, COL1A1, HMOX1, THBS1, and GPT) (Fig. 7C). To elucidate upstream regulatory mechanisms of the nine hub genes, transcription factor (TF)-target networks were constructed, showing that KLF16 emerged as the core transcriptional regulator (Fig. 7D). Heatmap analysis of the nine hub genes in the GSE199939 transcriptomic dataset (DFU vs CON) revealed significant upregulation of IL1B, FN1, MMP9, CCL2, PTPRC, COL1A1, HMOX1, and THBS1, and downregulation of GPT in DFU patients (Fig. 7E). The diagnostic efficacy of the nine hub genes was evaluated using ROC curve analysis, which exhibited reasonable discriminatory capabilities in distinguishing DFU patients from healthy controls (Fig. 7F).

#### Single-cell landscape of DFU

**Figure 8.**
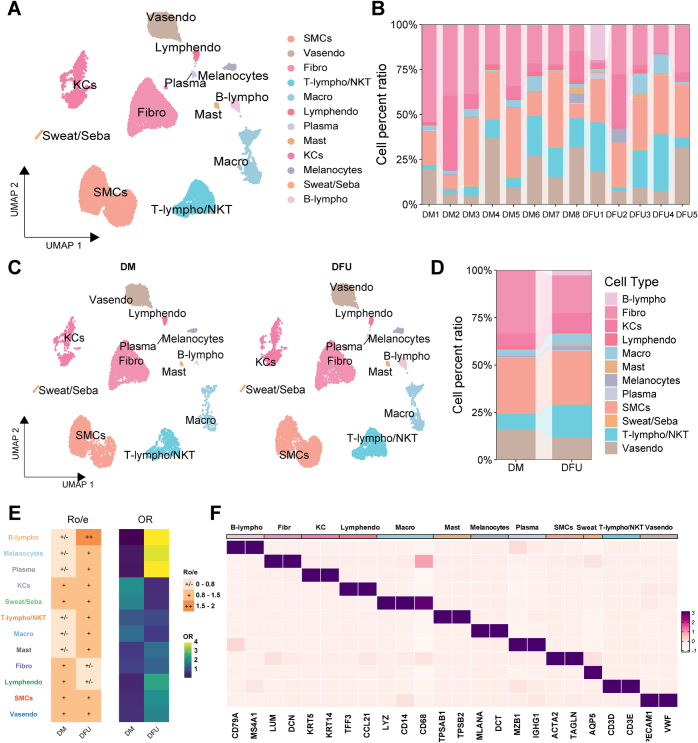
Single-cell transcriptomic profiling of DFU. (A) UMAP plots of 12 clusters in DFU and DM foot skin tissues, with each color indicating the associated cell type. (B) Relative percentages of cell populations in different sample sources. (C) UMAP plots in the DFU and DM groups, with each color indicating the corresponding cell type. (D) Relative percentages of cell populations in different group sources. (E) Heatmap showing the occurrence of cellular clusters with *R*_o/e_ and OR in each group. (F) Heatmap of marker gene expression. DFU, diabetic foot ulcer.

To systematically investigate the cellular interplay in DFU under varying Hcy conditions, we collected the GSE165816 dataset comprising eight DM foot skin tissues and five DFU tissues. After quality control and batch effect correction, we obtained high-quality transcriptome data from 17 102 cells, including 10 126 cells from DFU tissues and 6976 cells from DM foot skin tissues. UMAP clustering identified 12 distinct cell types (Fig. 8A, ), including fibroblasts (Fibro), smooth muscle cells (SMCs), keratinocytes (KCs), macrophages (Macro), B-lymphocytes (B-lympho), T-lymphocytes/NKT cells (T-lympho/NKT), plasma cells (Plasma), mast cells (Mast), lymphatic endothelial cells (Lymphendo), melanocytes (Melanocytes), vascular endothelial cells (Vasendo), and sweat/sebaceous gland cells (Sweat/Seba). Each cell cluster was annotated and categorized based on marker genes, and their expression patterns across different cell populations were visualized as heatmaps in Fig. 8F. To further explore tissue-level heterogeneity, we compared the proportional distribution of cell types at both individual (Fig. 8B) and inter-group levels (Fig. 8D). Statistical frameworks incorporating odds ratio (OR) and the ratio of observed to expected cell numbers (R_o/e_) revealed that B-lympho, plasma cells, and melanocytes demonstrated significant DFU association (Fig. 8E).

#### Single-cell profiling identifies Hcy-driven immune dysregulation and metabolic reprogramming in DFU

**Figure 9.**
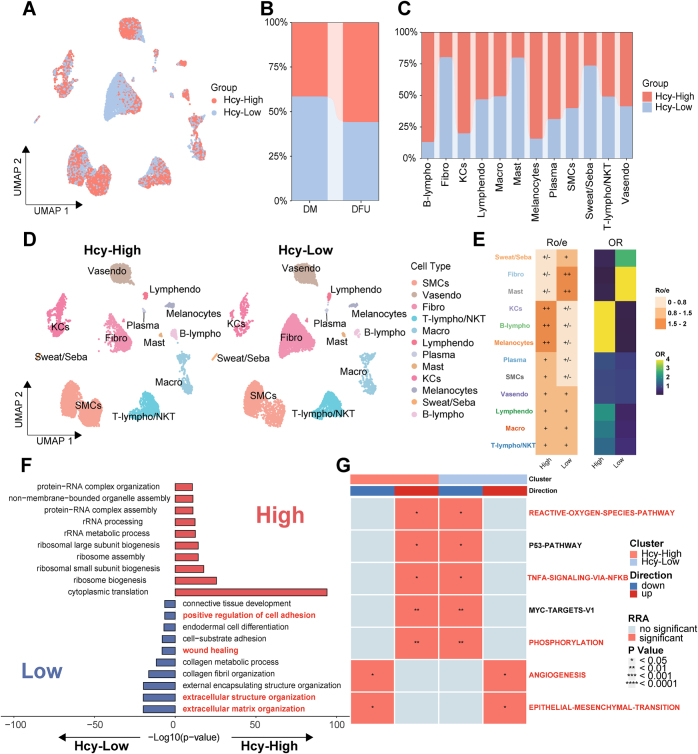
Single-cell transcriptomic profiling of DFU under varying Hcy conditions. (A) UMAP plots in Hcy-High and Hcy-Low groups, with each color indicating the corresponding cell type. (B) Relative percentages of Hcy-High and Hcy-Low groups in different group sources. (C) Cell type-specific proportions of Hcy-High and Hcy-Low cells across pooled DFU and DM samples. (D) UMAP plots in the Hcy-High and Hcy-Low groups. (E) Heatmap showing the occurrence of cellular clusters with R_o/e_ and OR in the Hcy-High and Hcy-Low groups. (F) Bar graphs of significantly activated pathways in high and low Hcy score groups (red and blue, respectively). (G) irGSEA analysis in high and low Hcy score groups. DFU, diabetic foot ulcer; GSEA, Gene Set Enrichment Analysis.

To establish a quantitative framework for assessing Hcy risk states, we used a parametric scoring system leveraging nine hub genes, enabling single-cell resolution evaluation of Hcy-related biological activity. We stratified cells into Hcy-High and Hcy-Low groups using the median threshold (Fig. 9A, ). Additionally, the proportion of Hcy-High cells was markedly increased in DFU tissues (Fig. 9B), indicating a potential mechanistic link between Hcy dysregulation and DFU progression. We further analyzed the proportion of Hcy-High cells across different cell types and tissues. Results revealed the greater prevalence of B-lympho, KCs, Melanocytes, Plasma, and Lymphendo in the Hcy-High group (Fig. 9C). Evaluation using the *R*_o/e_ index and OR confirmed stronger associations of B-lympho and KCs with the Hcy-High group (Fig. 9E). To validate these findings across tissue contexts, we performed subgroup analyses in DFU and DM samples separately. As shown in Supplemental Digital Content Figure S4, available at: http://links.lww.com/JS9/F425, Hcy-High states were consistently enriched in DFU tissues, particularly within B cells, KCs, melanocytes, and plasma cells, corroborating the trends observed in the pooled dataset. Interestingly, B cells and melanocytes also showed elevated proportions of Hcy-High cells in DM skin, potentially reflecting early-stage or systemic manifestations of Hcy dysregulation. Furthermore, by applying enrichment analyses, we showed that Hcy-Low cells were involved in “wound healing,” “positive regulation of cell adhesion,” “extracellular structure organization,” and “extracellular matrix organization” (Fig. 9F). Furthermore, pathway activity analysis using the irGSEA algorithm demonstrated that the Hcy-High group exhibited upregulation of “reactive-oxygen-species-pathway,” “oxidative phosphorylation,” and “TNFα-signaling-via-NFκB” (Fig. 9G).

**Figure 10.**
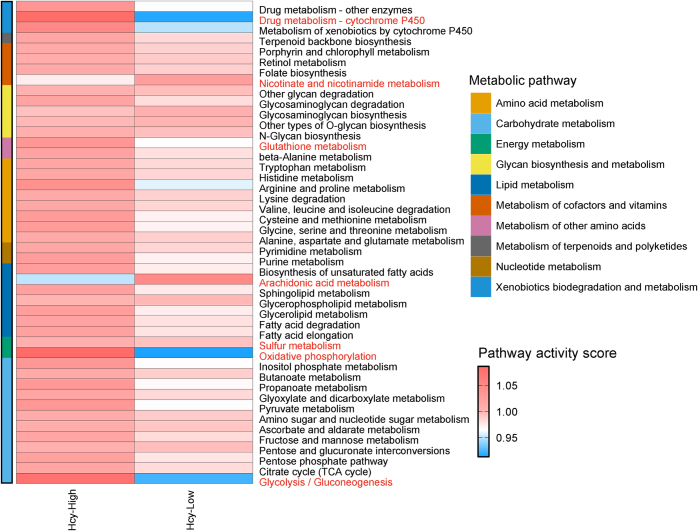
Metabolic reprogramming in Hcy-High and Hcy-Low groups.

Metabolic pathway analysis revealed profound reprogramming between Hcy-High and Hcy-Low cellular states (Fig. 10). The Hcy-High group exhibited global metabolic upregulation, including oxidative phosphorylation, glutathione cycling, and glycolysis/gluconeogenesis, reflecting adaptive responses to inflammatory stress through enhanced ATP production. Notably, the upregulation of drug metabolism enzymes in the Hcy-High group might explain the drug-resistant state of DFU cells. Compensatory Hcy clearance was evidenced by the activation of sulfur metabolism and the cysteine–methionine metabolism. In contrast, nicotinate/nicotinamide metabolism and arachidonic acid metabolism were enriched in the Hcy-Low group, which may support redox homeostasis and inflammation resolution.

#### Hcy reshapes the cellular interaction network

**Figure 11.**
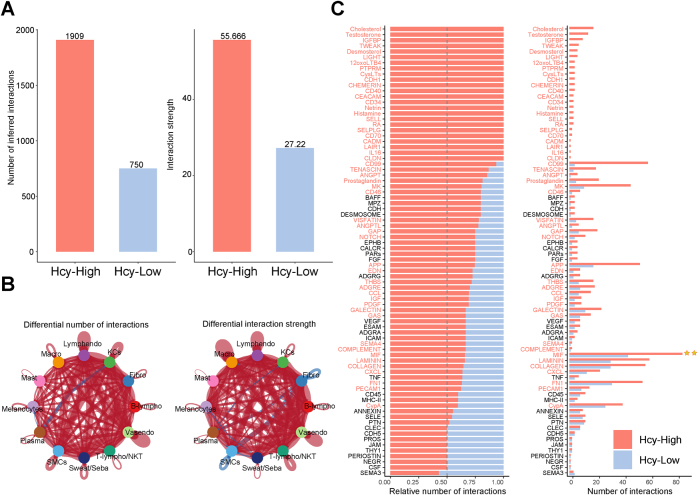
Characteristics of cell communication mediated in Hcy-High and Hcy-Low groups. (A) Bar plots of interaction numbers and strengths in Hcy-High and Hcy-Low groups. (B) Circle network plots of interaction numbers and strengths in Hcy-High and Hcy-Low groups. (C) Differences in signaling pathway intensities between Hcy-High and Hcy-Low groups.

Cellular crosstalk using CellChat revealed a significant overall increase in both the number and strength of interactions in the Hcy-High group (Fig. 11A-B). Several signaling pathways were notably enriched, including MIF, CD99, MK, APP, LAMININ, COLLAGEN, FN1, and CXCL, with MIF signaling exhibiting the highest activity (Fig. 11C).

**Figure 12.**
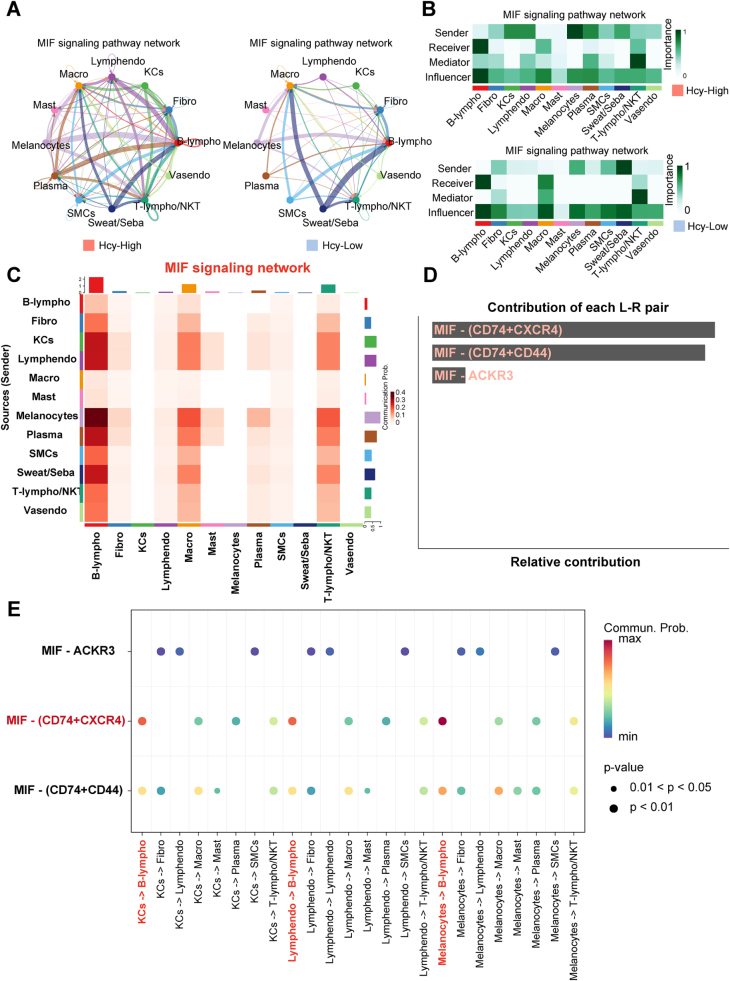
MIF-mediated cellular crosstalk in Hcy-High and Hcy-Low groups. (A) Circular plots comparing MIF signaling strength between Hcy-High and Hcy-Low groups. (B) Weighted roles of MIF signal senders, receivers, mediators, and influencers in each group. (C) Heatmap of MIF interaction probabilities in the Hcy-High group. (D) Activation levels of MIF-associated ligand-receptor pairs. (E) Cell-type-specific MIF signaling axes supporting stromal-B cell crosstalk.

In the Hcy-High group, the MIF axis was primarily sent by melanocytes, KCs, and Lymphendo, mediated by T-lympho/NKT, and received by B-lympho. In contrast, in the Hcy-Low group, Sweat/Seba emerged as the predominant signal senders, while the overall interaction strength was markedly reduced (Fig. 12A-C). Further analysis in the Hcy-High group identified three MIF-associated ligand–receptor pairs: MIF-CD74/CXCR4, MIF-CD74/CD44, and MIF-ACKR3, among which MIF-CD74/CXCR4 exhibited the highest activation level (Fig. 12D). Notably, this signaling axis exhibited the highest interaction probability in Melanocytes-B-lympho, KCs-B-lympho, and Lymphendo-B-lympho, supporting the hypothesis that Hcy activates the stromal cell-B-lympho axis via the MIF receptor complex (Fig. 12E).

### Association of T2D and Hcy levels with all-cause mortality

**Figure 13.**
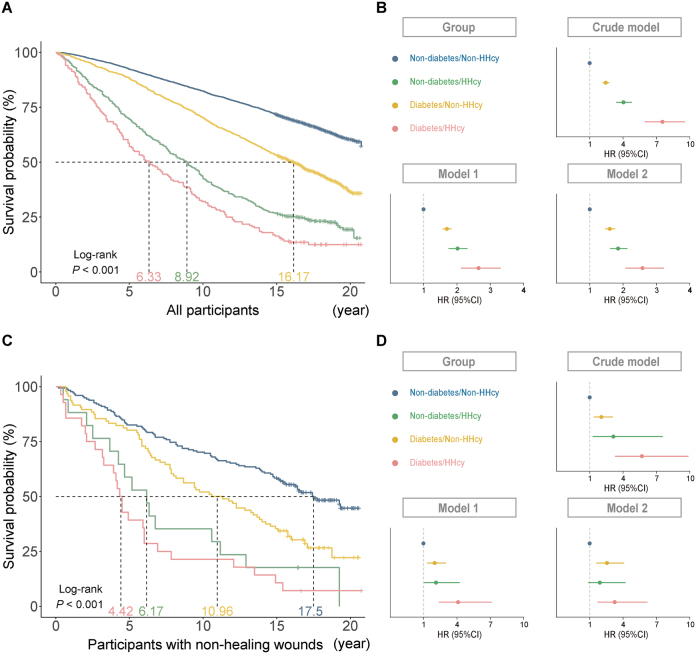
Association of T2D and HHcy with all-cause mortality in the overall population and participants with non-healing wounds. (A) Kaplan–Meier survival curves for the overall population. (B) Cox proportional hazards models for the overall population. (C) Kaplan–Meier survival curves for participants with non-healing wounds. (D) Cox proportional hazards models for participants with non-healing wounds. Kaplan–Meier survival analysis shows significant differences between the four groups: non-diabetes/non-HHcy, non-diabetes/HHcy, diabetes/non-HHcy, and diabetes/HHcy. The Cox proportional hazards models provide HR values for all four groups, adjusted for age, sex, race, marital status, smoking status, alcohol use, family PIR, BMI, vitamin B12, and methylmalonic acid. Dots: HRs; Horizontal lines: 95% CIs.

T2D and elevated Hcy levels were significantly associated with increased all-cause mortality in both the overall population and participants with non-healing wounds, as demonstrated by Kaplan–Meier survival curves and Cox proportional hazards regression analyses (Fig. 13; Supplemental Digital Content Table S9, available at: http://links.lww.com/JS9/F425). Kaplan–Meier survival curves revealed significant differences in survival across the four groups (log-rank *P* < 0.001), with the diabetes/HHcy group exhibiting the poorest survival outcomes in both populations (Fig. 13A, ). In adjusted Cox models, participants in the non-diabetes/HHcy, diabetes/non-HHcy, and diabetes/HHcy groups exhibited significantly higher risks of all-cause mortality compared to the non-diabetes/non-HHcy group in both the overall population [adjusted HRs (95% CIs): 1.85 (1.61–2.13), 1.60 (1.46–1.76), 2.58 (2.07–3.22)] and in participants with non-healing wounds [1.91 (0.86–4.22), 2.55 (1.59–4.08), 3.24 (1.70–6.20)] (Fig. 13B, ; Supplemental Digital Content Table S9, available at: http://links.lww.com/JS9/F425). No significant additive or multiplicative interactions between diabetes and elevated HHcy were observed concerning all-cause mortality (Supplemental Digital Content Table S10, available at: http://links.lww.com/JS9/F425).

In addition, Kaplan–Meier analysis confirmed that patients with a history of non-healing wounds had a higher risk of all-cause mortality (log-rank *P* < 0.001) (Supplemental Digital Content Figure S3, available at: http://links.lww.com/JS9/F425), emphasizing the long-term consequences of impaired wound healing.

## Discussion

This study recruited 8406 adults from a nationally representative US sample and found that both T2D and elevated Hcy levels independently increased the risk of non-healing wounds and all-cause mortality. Notably, we demonstrated for the first time a significant additive interaction between T2D and HHcy, highlighting their synergistic effect on non-healing wounds. In male diabetic patients, a J-shaped relationship between Hcy levels and non-healing wound risk was observed, with the lowest risk occurring at around 8.9 µmol/L. Additionally, systemic inflammatory markers, including RDW, MLR, SIRI, and NLR, were found to mediate the association between Hcy and non-healing wounds in diabetic patients. Integrative single-cell and bulk transcriptomic analysis identified nine pathologically significant Hcy-associated hub genes in DFU, enabling the first construction of a risk score system. This multi-omics approach mechanistically elucidated that Hcy drives DFU progression by inducing metabolic reprogramming and stromal-immune interactions. Finally, our results emphasized the long-term consequences of impaired wound healing. Collectively, these findings highlight Hcy as a crucial factor in impaired wound healing, offering valuable insights for risk stratification and targeted prevention strategies.

Chronic non-healing wounds result from complex interactions between various underlying comorbidities, including diabetes, metabolic disorders, obesity, vascular disease, lifestyle factors, and genetic predisposition. Elevated hyperglycemia (HG) and HHcy jointly damage endothelial cells and microvascular structures, leading to local hypoxia, inflammation, and a prothrombotic state, thus worsening wound healing. For example, Cheng *et al* confirmed that HG exacerbates HHcy, with both conditions synergistically inducing endothelial dysfunction through μ-calpain/PKCβ2 activation and eNOS inhibition^[44]^. In addition to vascular mechanisms, Fan *et al* highlighted the synergistic impact of T2D and HHcy on cardiovascular events, reporting hazard ratios of 1.85 for stroke, 1.78 for major adverse cardiovascular events, and 1.33 for myocardial infarction in diabetes/HHcy group^[45]^. However, these analyses relied solely on Cox regression models, without detailed interaction metrics such as RERI, AP, or SI to quantify the additive effects of these conditions. In our study, we expanded the focus to include non-healing wounds and identified that participants with both T2D and HHcy had the highest risk (OR: 5.28). Importantly, we observed a significant additive interaction between T2D and HHcy (RERI: 2.83, AP: 0.54, SI: 2.95), indicating that their combined effect was approximately three times the sum of their individual effects. This joint analysis provides robust evidence of the unique synergistic impacts of T2D and HHcy on non-healing wounds, offering valuable insights for risk prediction and targeted prevention strategies.

In diabetic males, a distinct J-shaped association was observed between Hcy levels and the risk of non-healing wounds. The lowest risk was observed at an Hcy level of 8.9 μmol/L, while both low and high Hcy levels were associated with an increased risk of wound non-healing. Sensitivity analyses confirmed that this phenomenon also applied to the general male population. Although most studies have focused on the detrimental effects of elevated Hcy, emerging evidence and our results indicate that low Hcy might also pose clinical risks. For instance, Plessis *et al* reported a U-shaped relationship between Hcy levels and blood pressure in adolescents, observing elevated risk in both the lowest and highest Hcy tertiles^[46]^. Moreover, 41% of 210 patients with Hcy levels below 6 μmol/L were diagnosed with idiopathic peripheral neuropathy (IPN), stressing the potential adverse consequences of low Hcy^[47]^. Metabolically, Hcy is a pivotal intermediate in the methionine (Met) cycle, maintaining DNA and protein methylation via remethylation and producing antioxidant molecules, such as glutathione and taurine, through the transsulfuration pathway. Thus, low Hcy may result from inadequate dietary methionine or from metabolic adaptations that prioritize glutathione synthesis under oxidative stress, leading to excessive depletion of Hcy stores and weakened repair and antioxidant defenses. In contrast, elevated Hcy exacerbates endothelial dysfunction and inflammation, thereby further impeding wound healing.

In contrast, we also found that women exhibited a positive linear correlation with the risk of non-healing wounds. Previous studies report that women typically have 20% lower Hcy levels, partly due to estrogen-mediated vascular protection that reduces oxidative stress and enhances endothelial function^[48–50]^. Consistent with this protective effect, diabetic men have a 1.5 times higher DFU risk and a significantly greater incidence of amputation than women^[4]^. Bertoia *et al* reported a strong link between elevated Hcy and PAD risk in men, but not in women^[10]^. These findings also suggest that, due to higher baseline Hcy levels and limited estrogen protection, men are more susceptible to the harmful effects of both low and high Hcy, explaining the observed J-shaped curve. In contrast, women may be relatively protected against low Hcy by estrogen, but as Hcy increases, their risk also rises.

Wound healing is influenced by sex-specific biological mechanisms, driven by immune regulation, hormonal regulation, and differences in Hcy metabolism. Women typically exhibit more active innate and adaptive immune responses, which can enhance wound healing but also increase susceptibility to autoimmune diseases^[51]^. In contrast, men are more prone to chronic low-grade inflammation. Estrogen, particularly 17β-estradiol, accelerates wound healing through multiple mechanisms. For instance, it downregulates neutrophil-expressed L-selectin, thereby reducing excessive neutrophil accumulation and elastase production in aged wounds, while simultaneously enhancing neutrophil phagocytic ability^[52,53]^. Additionally, Zhang *et al* demonstrated that 17β-estradiol upregulates cystathionine β-synthase (CBS) and cystathionine γ-lyase (CSE), promoting the conversion of Hcy into H_2_S, which alleviates Hcy toxicity^[54,55]^. Estrogen also activates the PI3K/Akt signaling pathway to mitigate oxidative stress and inflammatory responses induced by Hcy and reduces vascular endothelial cell pyroptosis via estrogen receptor α (ERα)-mediated autophagy activation^[56,57]^. Furthermore, estrogen directly promotes keratinocyte and fibroblast migration and indirectly accelerates re-epithelialization and collagen deposition by stimulating macrophage-derived platelet-derived growth factor (PDGF), contributing to improved tissue repair^[58]^. In contrast, wound repair is inhibited by male gonadal hormones such as testosterone and its metabolite 5α-dihydrotestosterone (DHT)^[59]^. Testosterone directly suppresses the healing response by increasing macrophage-driven pro-inflammatory cytokine expression. Castration in male mice significantly accelerates cutaneous wound healing, reduces local inflammation, and enhances hair growth^[60]^. These findings highlight the dual roles of sex hormones in the regulation of immune responses and wound repair, providing a mechanistic basis for the observed clinical disparities in wound healing outcomes between men and women.

We also found that RDW, MLR, NLR, and SIRI may mediate the relationship between elevated Hcy levels and diabetic non-healing wounds. While the underlying mechanisms remain unclear, several possible explanations exist. RDW elevation typically reflects impaired red blood cell function and is often associated with chronic inflammation, oxidative stress, and deficiencies in iron, folate, and vitamin B12^[61]^. B12 deficiency elevates Hcy levels, which in turn exacerbate inflammation, further depleting both B12 and iron through cytokine release. This cascade increases free radical production, impairing erythrocyte function and raising RDW. Peng *et al* found an independent association between RDW and Hcy levels^[62]^. Furthermore, for every 0.1 unit increase in RDW, the risk of PAD increases by 19%^[63]^. Therefore, elevated RDW may serve as a critical link between elevated Hcy levels and impaired wound healing. MLR and NLR reflect the inflammatory activity of monocytes and neutrophils, while SIRI assesses systemic inflammation^[64]^. Elevated Hcy intensifies systemic inflammation and vascular dysfunction by inducing inflammatory monocyte subsets, increasing the number of monocytes and macrophages in both lesion sites and peripheral tissues^[65,66]^. Gao *et al* reported significantly increased MLR and NLR in T2D patients with DFU^[67]^. These findings suggest that the increased inflammatory markers reflect a systemic inflammatory response and immune dysregulation induced by Hcy, contributing to impaired wound healing.

According to the Hcy risk scoring system, significant metabolic differences were observed between the Hcy-High and Hcy-Low groups. The nicotinate metabolic pathway was markedly inhibited in the Hcy-High group cells, leading to inadequate NAD⁺ synthesis, which weakened cellular antioxidant capacity and exacerbated DNA damage. This finding is consistent with clinical observations of delayed wound healing in HHcy patients. It was found that nicotinamide repairs mobilization impairment of endothelial progenitor cells and inhibits apoptosis under diabetic conditions by targeting NAD⁺ metabolic deficiencies^[68]^. Additionally, nicotinamide riboside combined with resveratrol synergistically modulates unfolded protein response homeostasis and downregulates NLRP3, caspase-1, and IL1B to suppress pyroptosis, thereby elevating intracellular NAD⁺ levels, improving energy metabolism, and ultimately accelerating diabetic rat wound healing^[69]^. These mechanisms suggest that Hcy-driven NAD⁺ depletion not only directly impairs endothelial function but also amplifies inflammatory cascades through oxidative stress, establishing a pathological foundation for aberrant activation of the MIF axis.

**Figure 14.**
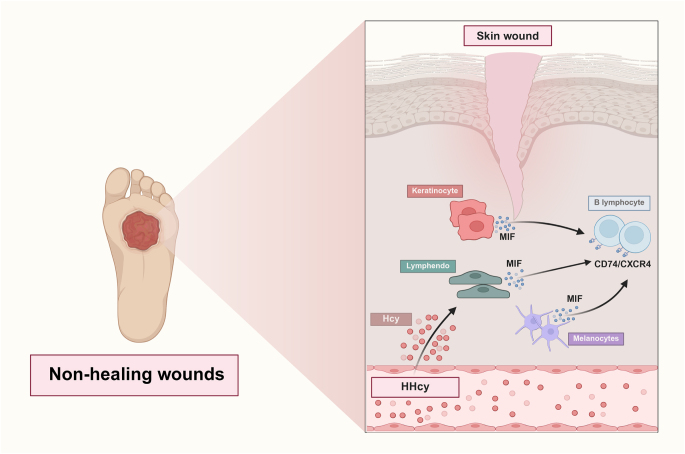
A mechanism summary figure. It was created by taking the template on BioRender.com as a reference, with permission.

Cellular communication analysis further revealed that the MIF-CD74/CXCR4 axis was identified as a central driver of stromal cell–B-lympho interactions in the Hcy-High group, implicating an imbalance in inflammatory responses. Macrophage migration inhibitory factor (MIF), a multifunctional cytokine in the immune system, not only regulates innate and adaptive immune responses but also affects endothelial homeostasis by mediating cell proliferation, differentiation, and apoptosis^[70,71]^. Hcy induces autophagy and apoptosis in HUVECs via activating the MIF/mTOR signaling pathway^[72]^. Serum MIF levels are significantly elevated in chronic wound patients compared to acute wound patients, further supporting its role in wound repair homeostasis dysregulation^[73]^. Nevertheless, the specific mechanisms by which the MIF-CD74/CXCR4 axis mediates inflammatory imbalance under high Hcy conditions remain to be fully elucidated in vitro and in vivo. To address this, future studies should prioritize Hcy stimulation experiments in fibroblasts or endothelial cells, with a comprehensive evaluation of NAD⁺ levels, oxidative stress markers, DNA damage, and cellular migration and repair capacity. Furthermore, cytokine profiling will help systematically characterize changes in inflammatory signaling associated with elevated Hcy. To clarify the functional roles of the MIF-CD74/CXCR4 axis, pathway inhibition studies using ISO-1 to block MIF activity and AMD3100 to inhibit CXCR4 should be carried out^[74,75]^. These integrated experimental approaches will be critical for revealing the molecular basis of Hcy-driven pathology (Fig. 14).

Our study has several strengths. First, we conducted this study in a large, nationally representative, multiethnic U.S. cohort, substantially improving the reproducibility and external validity of our findings. Second, to our knowledge, this is currently one of the largest and most comprehensive investigations of the individual and synergistic interactions between Hcy and T2D on non-healing wounds, while also assessing all-cause mortality as a long-term outcome. Third, the sex-specific J-shaped association observed in male patients underscores the necessity of tailored interventions and highlights the clinical significance of our findings. Fourth, our mediation analysis of clinically accessible inflammatory markers represents an innovative step in elucidating the mechanistic pathways by which elevated Hcy may induce vascular and immunological disturbances. Fifth, this work uses the concept of combining bioinformatics methods with epidemiological studies, consistent with the growing emphasis on precision medicine in epidemiology and translational science. Multiple stratification and sensitivity analyses bolster the reliability of our results.

This study offers several clinically relevant insights. First, the J-shaped association between Hcy and non-healing wound risk in diabetic men suggests that both elevated and low Hcy levels may be detrimental, calling for reconsideration of current Hcy reference ranges and more nuanced, sex-specific monitoring strategies. Future research should explore whether Hcy risk scoring models can be stratified or standardized by sex to account for these biological differences, potentially improving their predictive accuracy and clinical utility. Second, the observed synergistic interaction between T2D and HHcy implies that individuals with both conditions constitute a distinct high-risk subgroup. These patients may benefit from more intensive surveillance, early vascular assessment, and metabolic interventions, which could be explored as adjunctive therapies for wound prevention. Third, the mediating role of systemic inflammatory markers highlights the potential of integrating routine hematological indices into wound risk models. As these markers are readily available and cost-effective, they could enhance early risk stratification in primary care. Finally, the Hcy-based multi-omics signature developed in this study provides a novel framework for understanding the molecular mechanisms of impaired wound healing in diabetes. Rather than serving as a predictive tool, this score functions as a diagnostic and mechanistic classifier that links Hcy metabolism to immune activation and stromal remodeling. It may help identify molecular subtypes of diabetic wounds and guide future biomarker development or targeted therapeutic research within a precision medicine paradigm.

Some inherent issues need to be acknowledged. First, as an observational study, even with rigorous statistical approaches, we cannot confirm causality. Second, single-time measurements of Hcy and inflammatory markers may not capture fluctuations over time. Third, self-reported data and wound classification in the NHANES dataset are subject to recall bias, misclassification, and phenotypic heterogeneity, as detailed wound etiology and anatomical information were unavailable. This may dilute effect estimates and limit clinical interpretability. Fourth, although we adjusted for multiple covariates, there may remain residual or unmeasured confounding factors, such as psychosocial stress or occupational exposures. Fifth, although we systematically assessed both additive and multiplicative interactions between T2D and HHcy, a significant interaction was observed only on the additive scale for non-healing wounds. No significant multiplicative interaction was detected, and we also found no interaction between T2D and HHcy for all-cause mortality. These null findings may be related to limited statistical power, sample size, or the heterogeneity of wound phenotypes in the NHANES dataset. These potential interactions warrant further investigation in larger and better-characterized cohorts. Finally, although our integrative multi-omics analyses suggest that the MIF-CD74/CXCR4 axis may mediate Hcy-driven immune dysregulation and impaired wound healing, these findings are primarily based on computational inference and cross-sectional data. Direct experimental validation is needed to confirm these mechanistic links.

Given these limitations, future studies should incorporate clinically adjudicated wound classifications, imaging, or electronic health record (EHR)-based outcome measures, and longitudinal designs. Prospective trials, multicenter cohorts, and experimental models are also needed to validate our findings and clarify related underlying mechanisms.

## Conclusion

**Figure 15.**
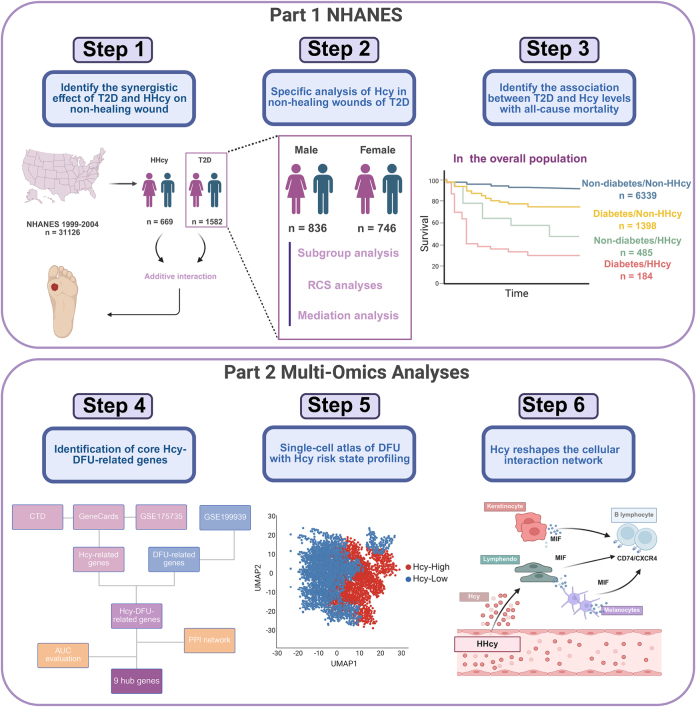
Study design and workflow of this study. It was created by taking the template on BioRender.com as a reference, with permission.

This study provides reliable evidence that elevated Hcy levels and T2D independently and synergistically increase the risk of non-healing wounds, with significant additive interaction. Notably, Hcy exhibits a sex-specific J-shaped association with non-healing wounds in male diabetic patients. Inflammatory mediators partially mediate the association between Hcy and impaired wound healing in diabetic individuals, while transcriptomic and single-cell analyses uncover Hcy-driven immune-metabolic reprogramming in DFU. Besides, the combined burden of T2D and HHcy significantly elevates all-cause mortality, particularly in individuals with non-healing wounds, emphasizing the long-term consequences of these comorbidities. This multi-omics framework positions Hcy as a central node in diabetic wounds, orchestrating metabolic dysregulation, inflammatory amplification, and cellular crosstalk (Fig. 15).

## Data Availability

Data will be made available upon reasonable request.

## Research registration unique identifying number (UIN)

This study is a retrospective analysis based on publicly available data from the National Health and Nutrition Examination Survey (NHANES). As NHANES data are de-identified and publicly accessible, ethical approval or review by an institutional review board is not required.
